# Electrophysiological evidence of severe marginal mandibular branch injury in Bell palsy: Implications for optimizing clinical assessment and treatment – a retrospective study

**DOI:** 10.1097/MD.0000000000049329

**Published:** 2026-06-19

**Authors:** Qiongfang Zhang, Mengjie Xia, Jinhuan Zhang, Yirong Chen, Zhihong Zou, Yongfeng Liu

**Affiliations:** a Department of Electromyography, Shenzhen Traditional Chinese Medicine Hospital, Shenzhen, Guangdong Province, China; b Department of Clinical Neurophysiology, Ruijin Hospital Affiliated to Shanghai Jiao Tong University School of Medicine, Shanghai, China; c Department of Acupuncture and Moxibustion, Shenzhen Traditional Chinese Medicine Hospital, Shenzhen, Guangdong Province, China.

**Keywords:** Bell palsy, electroneurography, facial nerve branches, marginal mandibular branch, nerve injury severity

## Abstract

Bell palsy (BP) is an acute idiopathic peripheral facial neuropathy typically presenting with unilateral weakness. However, the differential electrophysiological involvement of its 6 main branches remains poorly defined, limiting branch-level diagnosis and targeted management. This retrospective study aimed to characterize nerve conduction parameters across these branches, compare injury severity, and refine electrophysiological protocols to enhance clinical decision-making. This retrospective study enrolled 56 patients with BP. Compound muscle action potential (CMAP) amplitude and distal motor latency (DML) were measured for each facial nerve branch on both the affected side and unaffected side. Side-to-side comparisons were performed using paired-samples *t* tests or Wilcoxon signed-rank tests, as appropriate. Data are expressed as mean ± standard deviation or median (interquartile range). A *P* value <.05 was considered statistically significant. Compared to the US, the affected side demonstrated significantly reduced CMAP amplitudes and prolonged DMLs across all branches (all *P* < .001). The severity of axonal injury, indicated by CMAP amplitude reduction ratios, ranked as follows: marginal mandibular > zygomatic (nasal) > buccal > zygomatic (orbital) > temporal > cervical. The severity of demyelinating injury, reflected by DML prolongation ratios, ranked as: marginal mandibular > buccal > zygomatic (orbital) > zygomatic (nasal) > temporal > cervical. The marginal mandibular branch exhibits the most severe electrophysiological involvement in BP. These findings underscore its critical role in disease severity and support the prioritization of this branch in both diagnostic assessment and targeted therapeutic intervention.

## 1. Introduction

Bell palsy (BP) also known as idiopathic facial nerve palsy, accounts for approximately 75% of all acute facial nerve palsy cases.^[1]^ It has an annual incidence of 15 to 30 per 100,000 individuals,^[2]^ predominantly affecting adults aged 20 to 40 years with a slight male predominance. As a disorder involving the seventh cranial nerve (facial nerve), it is characterized by acute-onset, mostly unilateral facial muscle paralysis. Typical clinical manifestations include disappearance of frontal forehead lines, incomplete eyelid closure, deviation of the oral commissure, flattening of the nasolabial fold, and air leakage from the buccal cavity during exhalation.^[3]^

Although its exact etiology remains unclear, BP is hypothesized to result from the interaction between anatomical predispositions and multiple pathogenic factors, including viral infection, ischemia, inflammation, and hypersensitivity to cold stimuli.^[4]^ These factors collectively induce facial nerve ischemia and edema, which subsequently cause myelin sheath and axonal damage, ultimately leading to facial motor dysfunction and paralysis.^[5]^ Approximately 15% to 30% of patients develop residual sequelae of varying severity,^[6]^ which can significantly impair patients’ social interactions, occupational performance, and psychological well-being.^[7]^

The current clinical diagnosis and management of BP rely primarily on physical examination and visual rating using facial grading scales, most notably the House–Brackmann grading scale.^[8]^ However, this subjective approach leads to variability in the diagnosis, monitoring, and evaluation of facial paralysis, specifically its progression and treatment outcomes, resulting in low inter- and intra-rater reliability in patient assessments. Therefore, objective assessment methods that can quantify the severity of facial nerve palsy are essential for optimizing clinical decision-making.

Studies have shown that 85% of the facial nerve trunk consists of motor axons.^[9]^ Motor neuron signals received by the facial nerve are transmitted via these axons to control facial muscle contraction. Facial electromyography combined with nerve conduction velocity (NCV) testing is a key neurophysiological assessment method. This technique involves supramaximal electrical stimulation of ipsilateral motor nerve fibers and recording of the compound muscle action potential (CMAP) amplitude and distal motor latency (DML) from the muscles innervated by each facial nerve branch using surface recording electrodes.

CMAP amplitude reflects the responsiveness and synchronization of nerve fibers to electrical stimulation,^[10]^ while DML reflects the conduction function of the nerve.^[11]^ A percentage-based formula can be used to calculate the proportion of damaged nerve fibers, thereby quantifying the degree of demyelination and axonal injury in each facial nerve branch. As a preferred diagnostic modality for facial nerve injury, this combined electrophysiological approach enables objective evaluation of the severity, therapeutic efficacy, and prognosis of facial paralysis,^[12]^ and plays an irreplaceable role in the diagnosis, treatment, and rehabilitation of BP.

## 2. Patients and methods

### 2.1. Study population

A retrospective analysis was performed on 56 patients (outpatients and inpatients) treated at Shenzhen Hospital of Traditional Chinese Medicine, Affiliated to Guangzhou University of Chinese Medicine, between September 2024 and January 2025. All patients were clinically and electrophysiologically diagnosed with idiopathic facial palsy. The mean age was 38.32 years. After excluding patients with a disease duration exceeding 1 year, the mean time from symptom onset to 1st medical consultation was 29.44 days. Detailed demographic, clinical, and electrophysiological data were collected for all participants.

Facial nerve conduction studies were performed bilaterally (unaffected side [US] and affected side [AS]), yielding data from 112 temporal branches, 112 zygomatic branches (ocular region), 112 zygomatic branches (nasal region), 112 buccal branches, 72 marginal mandibular branches, and 72 cervical branches, with corresponding DML values recorded for each branch.

This study was conducted in accordance with the Declaration of Helsinki and approved by the Medical Ethics Committee of Shenzhen Hospital of Traditional Chinese Medicine (Fourth Clinical Medical College of Guangzhou University of Chinese Medicine; approval No. K2025-136-01).

## 3. Inclusion and exclusion criteria

### 3.1. Inclusion criteria

Patients were enrolled if they met the diagnostic criteria for idiopathic facial nerve palsy as defined by de Almeida JR et al^[13]^ and presented with the following clinical manifestations: unilateral onset of symptoms, including drooping of the oral commissure, facial asymmetry with deviation of the mouth and eye toward the US, widened palpebral fissure and epiphora on the AS, loss of frontal wrinkles, stiffness and numbness of the facial muscles, flattened nasolabial fold, and retroauricular pain. Additional inclusion criteria were as follows: unilateral, acute or subacute onset; disease duration of 5 to 7 days from symptom onset; no previous surgical intervention for facial palsy.

### 3.2. Exclusion criteria

Patients were excluded if they had any of the following conditions as described by Somasundara D et al^[14]^: upper motor neuron lesions (e.g., central facial palsy), auricular herpes zoster, space-occupying lesions (e.g., acoustic neuroma and parotid gland tumor), autoimmune diseases (e.g., Guillain-Barré syndrome), sarcoidosis, a history of jugular vein catheterization, or a history of cardiac pacemaker implantation.

## 4. Methodology

A comprehensive retrospective review was performed on 56 patients diagnosed with BP. All patients were confirmed by combined clinical evaluation and electrophysiological examination and received treatment at the inpatient and outpatient departments of Shenzhen Hospital of Traditional Chinese Medicine, Affiliated to Guangzhou University of Chinese Medicine, between September 2024 and January 2025.

The present study was based on the classic classification system of the 5 extracranial branches of the facial nerve. According to their distinct motor innervation territories, the zygomatic branch was further subdivided into 2 independent subbranches: the zygomatic branch (ocular region) and the zygomatic branch (nasal region).

The primary study endpoints included the DML and CMAP amplitude of 6 target facial nerve branches on both the AS and contralateral US: the temporal, zygomatic (ocular region), zygomatic (nasal region), buccal, marginal mandibular, and cervical branches.

Standardized quantitative electrophysiological assessments were performed for all 6 branches, ensuring accurate evaluation of regional facial nerve function. Based on these measurements, paired comparative statistical analyses were conducted to compare electrophysiological parameters between the AS and US.

The technical procedures for nerve conduction studies and data collection standards were fully in accordance with the guidelines for facial nerve motor function testing described in *Electromyography and Neuromuscular Diseases: From Clinical to Electrophysiology*.^[15]^

### 4.1. Testing method

All facial nerve conduction studies were performed in a quiet, temperature-controlled room (25°C–30°C) using a Nicolet VikingQuest Electrodiagnostic System (Natus Neurology Incorporated). Before testing, the target skin areas were thoroughly cleaned to reduce skin impedance and ensure stable electrode-skin contact.

**Figure 1.**
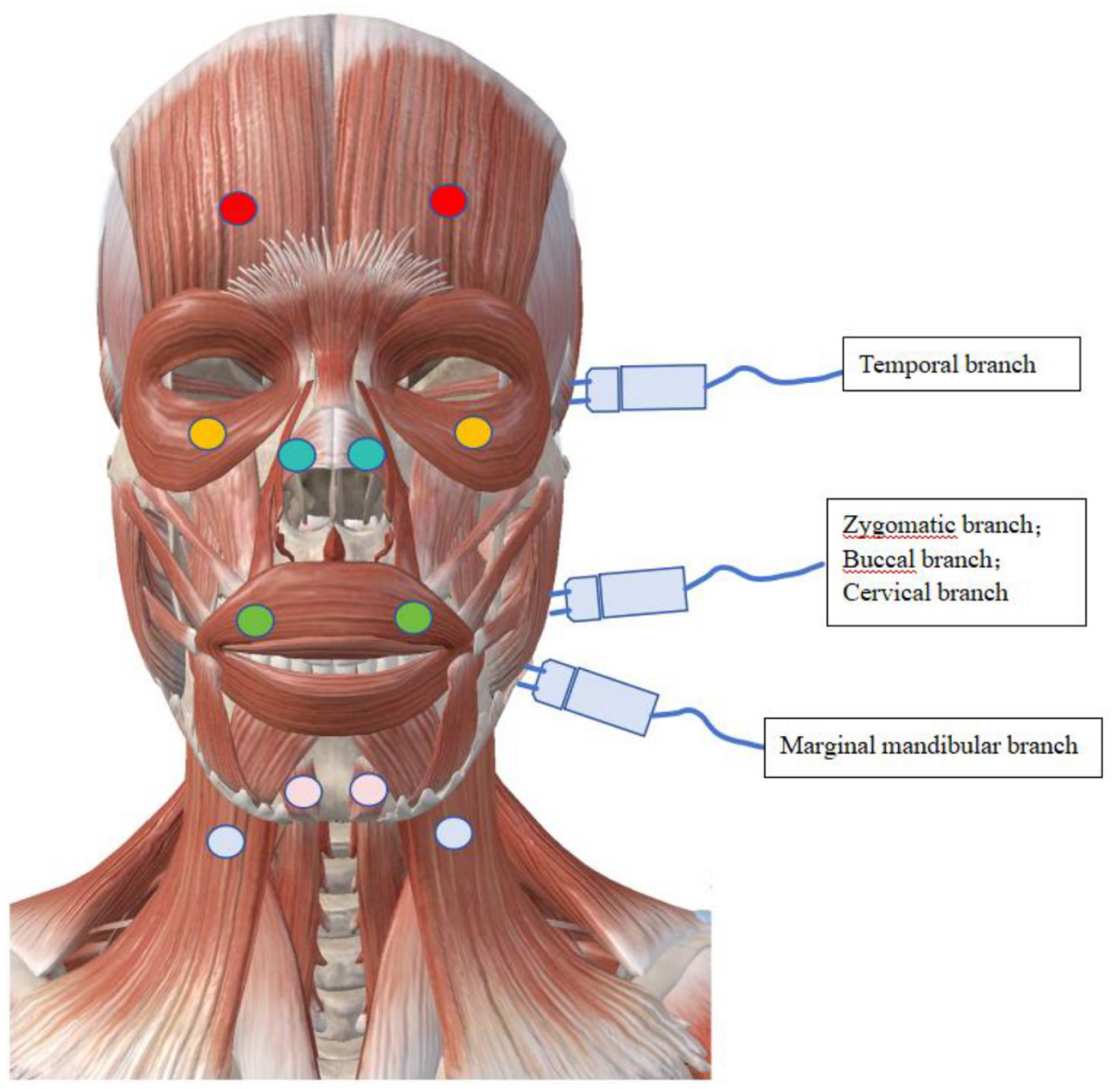
Placement of recording, reference, and stimulating electrodes. Color-coded correspondence between facial nerve branches and target muscles: red: frontalis muscle – temporal branch; yellow: orbicularis oculi muscle – zygomatic branch (orbital portion); blue: nasalis muscle – zygomatic branch (nasal portion); green: orbicularis oris muscle – buccal branch; pink: mentalis muscle – marginal mandibular branch; light blue: platysma muscle – cervical branch. See also Table 1 for detailed protocols.

The placement of stimulating, recording, and reference electrodes for each of the 6 facial nerve branches was standardized according to Figure 1. Detailed anatomical landmarks and nerve-muscle matching are presented in Table 1.

**Table 1:**
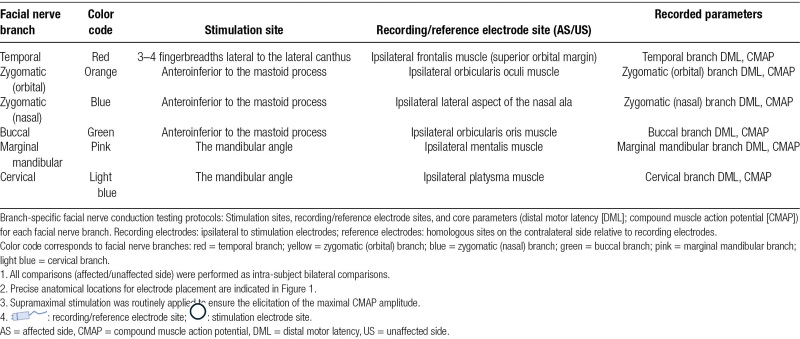
Branch-specific testing protocols for facial nerve branches.

Electrical stimulation was initiated at 1 mA and gradually increased until the CMAP amplitude plateaued (i.e., no further increase with additional current). Stimulation intensity was then increased by 10% above the plateau level to achieve supramaximal stimulation, thereby ensuring complete activation of all motor nerve fibers and reliable recording of maximal CMAP amplitudes.

The main electrophysiological parameters recorded for each branch were DML and CMAP amplitude. DML represents the conduction time from stimulation to the onset of the muscle response, reflecting the conduction velocity of the nerve fibers. CMAP amplitude corresponds to the synchronized summation of action potentials from all activated muscle fibers innervated by the target branch, reflecting the number of functioning motor units.

Normal reference values for DML and CMAP, together with color-coding corresponding to Figure 1, are summarized in Table 1 to ensure standardized testing and consistent data interpretation.

Strict quality control was applied throughout all examinations:

· Stimulation intensity was increased gradually to avoid muscle contraction artifacts and tissue irritation.
· A quiet testing environment was maintained to minimize electromagnetic interference.
· Supramaximal stimulation was used for all recordings to guarantee the accuracy and reproducibility of electrophysiological data.

### 4.2. Statistical methods

Electrophysiological data from eligible patients enrolled between September 2024 and January 2025 were extracted and organized using Microsoft Excel. All statistical analyses were performed using IBM SPSS Statistics version 27.0 (IBM Corp., Armonk). Parameters assessed included CMAP amplitude, DML, motor conduction velocity, and *F* wave, obtained from both the US and AS.

For missing CMAP amplitude values on the AS, a value of 0.0009 mV was imputed, corresponding to the minimum detectable limit of 0.001 mV for the recording system. Missing DML values on the AS were imputed using a rank-based adjustment: 0.2 ms was added to the maximum observed DML value in the study cohort.

Normality testing was performed for all variables. Normally distributed data were presented as mean ± standard deviation (SD) and compared using paired-samples *t* tests. Non-normally distributed data were presented as median (interquartile range) and analyzed using paired Wilcoxon signed-rank tests.

Asymmetry ratios between the US and AS were calculated to quantify the severity of facial nerve injury:

1. CMAP amplitude reduction ratio (%) = [(US CMAP − AS CMAP)/US CMAP] × 100%
2. DML prolongation ratio (%) = [(AS DML − US DML)/US DML] × 100%

**Figure 2.**
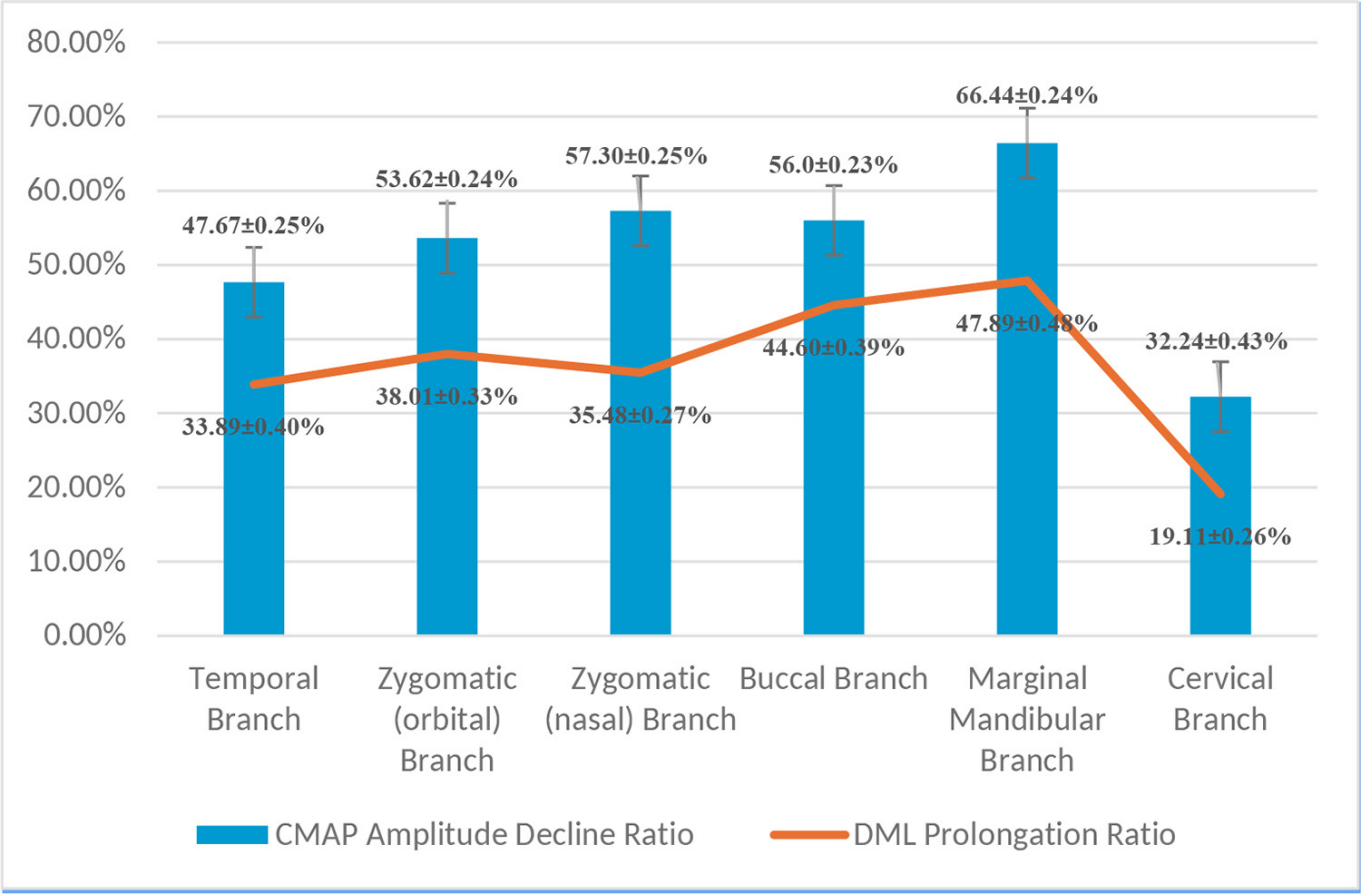
CMAP amplitude reduction and DML prolongation ratios of facial nerve branches in patients with BP: affected versus US. Data are presented as mean ± standard deviation (%). Sample size (n): 56 for the temporal, zygomatic (orbital), zygomatic (nasal), and buccal branches; 36 for the marginal mandibular and cervical branches. CMAP amplitude reduction ratios across facial nerve branches (descending order): marginal mandibular > zygomatic (nasal) > buccal > zygomatic (orbital) > temporal > cervical. DML prolongation ratios across facial nerve branches (descending order): marginal mandibular > buccal > zygomatic (orbital) > zygomatic (nasal) > temporal > cervical. BP = Bell palsy, CMAP = compound muscle action potential, DML = distal motor latency, US = unaffected side.

All ratio data were expressed as mean ± SD and summarized in Table 6 and Figure 2.

#### 4.2.1. Sample size estimation

Sample size calculation was performed using G*Power software (version 3.1.9.7; Heinrich-Heine-Universität Düsseldorf, Düsseldorf, Germany) with the following settings:

- Effect size (Cohen *f*): A large effect size (*f* = 0.4) was adopted based on preliminary pilot data, which demonstrated statistically significant and clinically relevant differences in electrophysiological parameters between the AS and US.
- Significance level (α): 0.05 (two-tailed)
- Statistical power (1 − β): 0.90
- Number of repeated measurements (*k*): 6 facial nerve branches
- Intraclass correlation coefficient: 0.4 (moderate within-patient correlation among branches)

The sample size was calculated using the formula for repeated measures ANOVA:


$$\begin{array}{c} n=\frac{{\left(Z_{1-\alpha /2\;}+Z_{1-\beta }\right)}^{2}\times \left(1-\rho \right)}{k\times f^{2}}. \end{array}$$

where:

- *Z*_1*−*α/2_ and *Z*_1−β_ are the critical values corresponding to the significance level and statistical power, respectively;
- *ρ* is the intraclass correlation coefficient;
- *k* is the number of repeated measurements (facial nerve branches);
- *f* is the effect size (Cohen *f*).

The minimum required sample size was 26 patients. To improve adjustment for potential confounding factors including age and disease duration, the final sample size was increased to 56 patients, consistent with recommended sample sizes for covariate adjustment (50–60 subjects).

## 5. Results

Normality testing was performed on the differences in electrophysiological parameters between the AS and US for all 6 facial nerve branches. The results of the normality tests determined the choice of statistical tests as follows:

Normally distributed data (analyzed using paired-samples *t* tests, presented as mean ± SD):

- Differences in CMAP amplitude for the zygomatic (nasal), marginal mandibular, and cervical branches (Table 2).
- Differences in DML for the temporal, zygomatic (orbital), and zygomatic (nasal) branches (Table 4).

**Table 2:** Differential analysis of CMAP amplitude between affected and unaffected sides of the zygomatic (nasal) branch, marginal mandibular branch, and cervical branch (mean ± standard deviation, $\bar{x}\pm S$).

Nerve branch	Affected side $\bar{x}\pm \;S$)		Unaffected side ($\bar{x}\pm \;S$)	n	*t*	*P*
Zygomatic branch (nose)		0.77 ± 0.48	1.86 ± 0.66	56	−12.264	<.001
Mandibular marginal branch		0.78 ± 0.63	2.28 ± 0.98	36	−10.482	<.001
Cervical branch		0.83 ± 0.55	1.28 ± 0.65	36	−5.304	<.001

Normality tests were performed on the CMAP amplitude differences between the unaffected and affected sides of the zygomatic (nasal) branch, marginal mandibular branch, and cervical branch in patients with facial paralysis. The results showed that *P > *.05, indicating that the differences conformed to a normal distribution. Therefore, paired-samples *t*-tests were used to analyze the inter-side CMAP amplitude differences, with results expressed as mean ± standard deviation.$\;\bar{x}\pm S$ All *P* values <.001, suggesting statistically significant inter-side differences.

CMAP = compound muscle action potential.

These differences were not statistically significant (all *P *> .05).

Non-normally distributed data (analyzed using paired Wilcoxon signed-rank tests, presented as median [interquartile range]):

- Differences in CMAP amplitude for the temporal, zygomatic (orbital), and buccal branches (Table 3).
- Differences in DML for the buccal, marginal mandibular, and cervical branches (Table 5).

**Table 3:** Differential analysis of compound muscle action potential (CMAP) amplitude between affected and unaffected sides of the temporal branch, zygomatic (orbital) branch, and buccal branch (median [25th, 75th percentiles]$M\left(P_{25},P_{75}\right)$).

Nerve branch	Affected side ($M\left(P_{25},P_{75}\right)$)	Unaffected side ($M\left(P_{25},P_{75}\right)$)	n	*Z*	*P*
Temporal branch	0.60 (0.40, 1.28)	1.40 (1.00, 2.23)	56	−6.518	<.001
Zygomatic (orbital) branch	1.05 (0.50, 1.60)	2.10 (1.90, 3.08)	56	−6.514	<.001
Buccal branch	0.90 (0.43, 1.48)	1.90 (1.53, 3.38)	56	−6.512	<.001

Normality tests were performed on the CMAP amplitude differences between the unaffected and affected sides of the temporal branch, zygomatic (orbital) branch, and buccal branch in facial paralysis patients. The results showed *P *< .05, indicating that the differences exhibited a non-normal distribution. Thus, the Wilcoxon signed-rank test was applied for statistical analysis (test statistic: *Z*-value), with data expressed as median (25th, 75th percentiles)$M\left(P_{25},P_{75}\right)$. All *P* values <.001, suggesting statistically significant inter-side differences.

**Table 4:** Differential analysis of distal motor latency (DML) between affected and unaffected sides of the temporal branch, zygomatic (orbital) branch, and zygomatic (nasal) branch (mean ± standard deviation, ($\bar{x}\pm \;S$).

Nerve branch	Affected side ($\bar{x}\pm \;S$)	Unaffected side ($\bar{x}\pm \;S$)	n	*t* value	*P* value
Temporal branch	3.91 ± 1.03	3.00 ± 0.63	56	7.230	<.001
Zygomatic (orbital) branch	4.09 ± 0.97	3.00 ± 0.51	56	8.837	<.001
Zygomatic (nasal) branch	4.26 ± 0.98	3.18 ± 0.65	56	10.288	<.001

Normality tests were performed on the DML differences between the unaffected and affected sides of the temporal branch, zygomatic (orbital) branch, and zygomatic (nasal) branch in facial paralysis patients. The results showed *P* > .05, indicating that the differences conformed to a normal distribution. Thus, the paired-samples *t* test was adopted for statistical analysis, with data expressed as mean ± standard deviation.$\bar{x}\pm S$ All *P* values <.001, suggesting statistically significant inter-side differences.

**Table 5:** Differential analysis of distal motor latency (DML) between affected and unaffected sides of the buccal branch, marginal mandibular branch, and cervical branch (median [25th, 75th percentiles]$M\left(P_{25},P_{75}\right)$).

Nerve branch	Affected side ($M\left(P_{25},P_{75}\right)$)	Unaffected side ($M\left(P_{25},P_{75}\right)$)	n	*Z*	*P*
Buccal branch	4.15 (3.40, 5.18)	3.00 (2.50, 3.50)	56	−6.471	<.001
Marginal mandibular branch	3.60 (2.85, 4.68)	2.70 (2.10, 3.10)	36	−4.813	<.001
Cervical branch	2.05 (1.83, 2.90)	1.90 (1.60, 2.28)	36	−4.412	<.001

Normality tests were performed on the DML differences between the unaffected and affected sides of the buccal branch, marginal mandibular branch, and cervical branch in facial paralysis patients. The results showed *P* < .05, indicating that the differences exhibited a non-normal distribution. Thus, the Wilcoxon signed-rank test was applied for statistical analysis (test statistic: *Z*-value; data expressed as median [25th, 75th percentiles] [*M*$\;\left(P_{25},P_{75}\right)$]). All *P* values <.001, suggesting statistically significant inter-side differences.

These differences were statistically significant (all *P* < .05).

As shown in Tables 2 and 3, the CMAP amplitudes of all 6 facial nerve branches on the AS were significantly lower than those on the US. Tables 4 and 5 demonstrate that the DML values of all 6 facial nerve branches on the AS were significantly prolonged compared with those on the US.

As summarized in Table 6 and Figure 2, the CMAP amplitude reduction ratio (a marker of axonal injury severity) across the facial nerve branches followed a descending order: marginal mandibular branch > zygomatic (nasal) branch > buccal branch > zygomatic (orbital) branch > temporal branch > cervical branch.

**Table 6:** Analysis of the difference ratio of CMAP amplitude and DML between unaffected and affected sides of facial nerve branches (mean ± standard deviation, $\bar{x}\pm \;S$).

Index	Temporal branch	Zygomatic (orbital) branch	Zygomatic (nasal) branch	Buccal branch	Marginal mandibular branch	Cervical branch
CMAP amplitude reduction ratio	47.67 ± 0.25	53.62 ± 0.24	57.30 ± 0.25	56.0 ± 0.23	66.44 ± 0.24	32.24 ± 0.43
DML prolongation ratio	33.89 ± 0.40	38.01 ± 0.33	35.48 ± 0.27	44.6 ± 0.39	47.89 ± 0.48	19.11 ± 0.26
n	56	56	56	56	36	36

Normality tests were performed to confirm that the difference ratios of CMAP amplitude and DML between the unaffected and affected sides of facial nerve branches in facial paralysis patients conformed to a normal distribution (*P* > .05). Thus, the paired-samples *t* test was used for statistical analysis, with results expressed as mean ± standard deviation. $\bar{x}\pm S$.

CMAP = compound muscle action potential, DML = distal motor latency.

Conversely, the DML prolongation ratio (a marker of demyelinating injury severity) was ranked as follows: marginal mandibular branch > buccal branch > zygomatic (orbital) branch > zygomatic (nasal) branch > temporal branch > cervical branch.

## 6. Discussion

### 6.1. Pathophysiological underpinnings of facial nerve motor control: relevance to clinical outcomes in BP

The motor network of the facial nerve is characterized by high anatomical specificity, which directly governs the complexity of facial muscle function and the prognostic trajectory of recovery in patients with BP. This anatomical specificity carries substantial implications for clinical diagnosis and treatment decision-making. Two key anatomical features are particularly relevant to the clinical management of this condition.

First, facial muscles are nearly devoid of muscle spindles.^[16]^ This unique structural trait means that motor regulation of facial musculature relies entirely on direct innervation from motor axons of the facial nerve. Consequently, even mild axonal or myelin injury can markedly impair muscle activation, highlighting the critical need for sensitive diagnostic tools to detect early neural dysfunction before overt clinical deterioration.

Second, the density of motor axons innervating facial muscles is approximately twice that of axons innervating intrinsic hand muscles,^[17]^ yielding a distinctive pattern of high neural innervation density relative to low muscle volume.^[18]^ Individual motor neurons innervate only a small subset of facial muscle fibers, supporting a fine-tuned regulatory architecture that enables precise control of facial expressions. This structural organization indicates that functional recovery from facial paralysis cannot be generalized across all facial regions. Instead, targeted evaluation of the 5 main extracranial facial nerve branches – temporal, zygomatic, buccal, marginal mandibular, and cervical – and branch-specific interventions for their corresponding target muscles are consistent with the goal of precision medicine in modern facial nerve management.

### 6.2. Neuroelectrophysiological testing: a cornerstone of objective assessment in BP

Under physiological conditions, muscle fibers belonging to intact motor units maintain a stable resting membrane polarization. When the facial nerve trunk is stimulated via standard neuroelectrophysiological approaches, afferent impulses propagate distally along the peripheral axons to innervate target facial myofibers, eliciting synchronous depolarization and measurable compound action potentials.

Quantitative analysis of CMAP amplitude and DML from innervated facial muscles enables clinicians to objectively evaluate the functional integrity of the facial nerve.^[19]^ This approach effectively overcomes inherent limitations of conventional subjective grading systems, such as the House–Brackmann grading scale, which rely heavily on visual inspection and clinical judgment, leading to inter-observer variability.

Specifically, side-to-side comparison of CMAP amplitudes and DML between the AS and US permits reliable stratification of neural injury severity. A more pronounced reduction in CMAP amplitude indicates more extensive axonal loss or dysfunction, whereas a greater prolongation in DML reflects a wider distribution of demyelinating injury – 2 key pathological features that underpin the clinical heterogeneity of BP.

Such objective neurophysiological quantification carries substantial clinical significance, not only for early and accurate diagnosis but also for risk stratification, personalized therapeutic decision-making (e.g., conservative treatment vs surgical decompression), and prognostication of long-term functional recovery. Collectively, these advantages establish neuroelectrophysiological testing as an indispensable tool in the contemporary clinical management of BP, aligning with the precision-driven approach highlighted in the preceding section.

### 6.3. Assessment and management of the marginal mandibular branch: an overlooked target in the diagnosis and treatment of BP

Current electrophysiological evaluation of the facial nerve in patients with BP remains disproportionately focused on the zygomatic and buccal branches, mainly because dysfunction in these 2 branches is most conspicuous during routine clinical examination. Although several authors have advocated inclusion of the temporal branch in standard assessment protocols,^[20]^ and pediatric studies have verified that the marginal mandibular branch may also be affected,^[21]^ this branch continues to represent a major blind spot in the clinical evaluation of adult patients and has not been integrated into mainstream diagnostic or therapeutic algorithms.

This diagnostic neglect carries meaningful clinical consequences. In patients presenting with prominent lower facial deficits – including labial ptosis and dysarthria – failure to examine the marginal mandibular branch frequently results in incomplete characterization of neural injury and erroneous stratification of injury severity. Such oversights may subsequently lead to suboptimal therapeutic planning, which directly undermines the principles of comprehensive and precise facial nerve management.

The distinct anatomical characteristics of the marginal mandibular branch predispose it to heightened vulnerability in BP, a feature with important clinical implications for diagnostic and therapeutic decision-making.

After traveling 15 to 20 mm within the parotid gland, this branch runs superficially along the inferior border of the mandible before innervating the lower lip musculature and the mentalis muscle.^[22]^ These muscles mediate essential physiological functions, including facial expression, speech articulation, and oral competence.^[23]^ Because of its superficial course and the absence of robust soft-tissue shielding, the marginal mandibular branch is particularly susceptible to the primary pathological processes underlying BP – viral inflammation and mechanical compression – leading to compressive injury or inflammatory damage secondary to tissue edema.^[24]^

Dysfunction of the marginal mandibular branch manifests clinically as paralysis or weakness of the lower lip and mentalis muscles^[25]^ and is frequently associated with an unfavorable long-term prognosis. This association is not incidental: such deficits typically reflect extensive neural injury, including widespread axonal loss. Accordingly, these findings reinforce the critical need to include the marginal mandibular branch in routine clinical and electrophysiological assessment.

### 6.4. Novel findings of this study: rethinking branch-specific injury in BP

Electrophysiological assessments in the present cohort revealed a statistically significant difference in injury severity across the facial nerve branches (*P *< .01), which challenges long-standing clinical paradigms and carries direct implications for the clinical evaluation of facial nerve function in BP. Two key novel observations emerged from our analyses:

First, the marginal mandibular branch exhibited the most severe axonal injury, with a mean CMAP amplitude reduction of 66.44% ± 0.24%, accompanied by prominent demyelinating changes (mean DML prolongation: 47.89% ± 0.48%). Notably, the injury severity of this branch was significantly greater than that of all other facial nerve branches.
Second, our findings contradict the traditional clinical consensus that the zygomatic and buccal branches are the most severely affected in BP. Instead, the marginal mandibular branch demonstrated significantly more pronounced damage compared with these 2 commonly prioritized branches.

Notably, these findings represent a meaningful paradigm shift, indicating that the current evaluation system centered on the zygomatic and buccal branches is incomplete and insufficient for comprehensive facial nerve assessment. Therefore, NCV examination of the marginal mandibular branch should be routinely incorporated into the standard electrophysiological workup for patients with BP. This strategy addresses an important unmet clinical need by enabling early detection and quantitative evaluation of lower facial nerve dysfunction, which constitutes a major source of patient morbidity, including functional impairment and psychosocial distress.

A key limitation of this study is its single-center, retrospective design with a relatively modest sample size (n = 56), which may increase the risk of selection bias and restrict the generalizability of the present findings.

### 6.5. Recommendations for optimizing clinical assessment: aligning tools with patient needs

Based on the findings of the present study, we recommend the adoption of the modified Sunnybrook grading system^[26]^ for the clinical evaluation of facial palsy. This recommendation is predicated on the system’s unique capacity to address a critical limitation of the widely utilized House–Brackmann grading scale: its inadequate assessment of lower facial motor function. Notably, the modified Sunnybrook scale explicitly incorporates evaluations of marginal mandibular branch function, including assessments of lower lip movement (e.g., symmetry during smiling or lip closure) and mentalis muscle activity (e.g., chin elevation and contraction).

Integration of this modified clinical grading scale with routine electrophysiological testing of the marginal mandibular branch – specifically, measurement of CMAP amplitude and DML – will facilitate a more comprehensive, objective, and precise assessment of facial nerve injury. This combined approach does not replace existing clinical and electrophysiological tools; rather, it enhances their clinical utility by ensuring systematic evaluation of both upper and lower facial motor function. Consequently, this integrated assessment strategy lays a solid foundation for the development of personalized treatment regimens that address the full spectrum of patient needs and align with the precision medicine principles highlighted in preceding sections.

### 6.6. Future research directions: advancing translational facial nerve care

#### 6.6.1. Multimodal assessment: integrating function and structure evaluations

Future investigations should aim to integrate quantitative electrophysiological parameters, including NCV and CMAP metrics, with high-resolution neuroimaging modalities. Specifically, the combination of electrophysiological testing with 3.0 T magnetic resonance facial nerve hydrography – for the detection of intraneural edema and compressive changes – and ultra-high-frequency ultrasound – for evaluating structural integrity of intracranial and extracranial nerve segments – may enable the establishment of a comprehensive function–structure dual-dimensional assessment system.

Electrophysiological indicators allow for precise quantification of motor dysfunction, including the degree of axonal injury and myelin integrity, whereas advanced neuroimaging permits direct visualization of anatomical abnormalities, such as nerve edema, compressed segments, and spatial relationships with adjacent structures, including the parotid gland and mandible. This multimodal approach overcomes the inherent limitations of single-modal examinations, which cannot concurrently provide functional quantification and anatomical localization. Accordingly, such an integrated strategy may furnish more robust, evidence-based support for clinical decision-making in the management of facial nerve injury.

#### 6.6.2. Prognostic prediction models: enabling evidence-based guidance for surgical decision-making

The development and clinical translation of prognostic prediction models represent a critical frontier in facial nerve research. Such models should incorporate 3 complementary domains of data: electrophysiological parameters (e.g., magnitude of CMAP amplitude reduction), high-resolution imaging features (e.g., severity of intraneural edema), and molecular biomarkers (e.g., serum levels of nerve growth factor and the proinflammatory cytokine interleukin-6).

Following rigorous validation in large-scale, multicenter clinical cohorts, these predictive models may be implemented as preoperative risk stratification tools to guide individualized surgical planning. For instance, patients classified as high-risk for unfavorable recovery may be prioritized for early aggressive interventions, such as facial nerve decompression combined with nerve repair scaffold implantation. Conversely, low-risk patients may be managed conservatively with serial dynamic monitoring. This paradigm supports risk-stratified personalized care, reduces unnecessary surgical interventions, and ultimately improves long-term functional outcomes.

#### 6.6.3. Electrophysiological typology: toward precision diagnosis and individualized therapy

Based on quantitative electrophysiological profiles – including the degree of CMAP amplitude reduction and DML prolongation – idiopathic BP and traumatic facial nerve injury can be subclassified into distinct pathophysiological subtypes: demyelination-dominant injury, axonal-dominant injury, and mixed injury.

This electrophysiological typing system permits the design of subtype-specific, integrated surgical and rehabilitation strategies. For patients with demyelination-dominant injury and preserved axonal integrity, minimally invasive facial nerve decompression may be considered as 1st-line therapy, followed by low-frequency electrical stimulation to facilitate remyelination. For those with severe axonal loss and near-absent CMAP responses, early nerve transfer procedures – such as deep temporal nerve to marginal mandibular branch transfer – may be warranted, in combination with individualized postoperative muscle reeducation to maximize functional recovery.

Further elucidation of the optimal therapeutic time window for each electrophysiological subtype will enable clinicians to implement interventions at the stage most conducive to neural repair and functional restoration.

#### 6.6.4. Optimizing therapeutic strategies for the marginal mandibular branch

The present observation that the marginal mandibular branch sustains disproportionately severe injury underscores the need for targeted diagnostic and therapeutic optimization to improve protection and repair of this often-neglected branch, a key objective in the future management of BP.

Several priority areas can be addressed. Conservative treatment should emphasize region-specific interventions targeted at the anatomical course of the marginal mandibular branch and the inferior lip depressor musculature. During facial nerve decompression surgery, the extent of extracranial decompression should be clearly defined with sufficient anatomical exposure to avoid incomplete decompression or missed injury, both common in conventional approaches. In nerve repair procedures, donor nerves with comparable functional properties, such as the platysma branch, should be preferentially selected, and intraoperative electrophysiological monitoring (e.g., real-time CMAP recording) may be used to confirm the functional integrity of neurorrhaphy, ensuring both anatomical accuracy and functional efficacy.

Future multicenter clinical trials are warranted to compare functional outcomes – including oral competence, amplitude of mouth excursion, and recovery rate of lower facial motion – between optimized and conventional protocols for the marginal mandibular branch. Such investigations will provide high-level evidence to refine surgical techniques and fulfill the core clinical goal of simultaneous functional and aesthetic restoration in facial nerve reconstruction.

## Author contributions

**Conceptualization:** Qiongfang Zhang, Yongfeng Liu.

**Data curation:** Qiongfang Zhang, Jinhuan Zhang, Yongfeng Liu.

**Formal analysis:** Qiongfang Zhang, Mengjie Xia, Jinhuan Zhang, Yirong Chen, Zhihong Zou.

**Funding acquisition:** Qiongfang Zhang.

**Methodology:** Qiongfang Zhang, Mengjie Xia, Jinhuan Zhang, Yirong Chen.

**Resources:** Qiongfang Zhang, Zhihong Zou.

**Writing – original draft:** Qiongfang Zhang, Mengjie Xia, Yongfeng Liu.

**Writing – review & editing:** Qiongfang Zhang, Mengjie Xia, Jinhuan Zhang, Yirong Chen, Yongfeng Liu.
